# Time to Make Sense: Doctors’ and Students’ Experiences of Difficult Feedback

**DOI:** 10.5334/pme.1689

**Published:** 2025-09-01

**Authors:** Leonie Griffiths, Joanne Hilder, Elizabeth Molloy, Anna Ryan, Christopher Watling

**Affiliations:** 1 The University of Melbourne, AU; 2 Southern Cross University, AU; 3 The Royal College of Physicians and Surgeons of Canada, CA

## Abstract

**Introduction:**

Learners’ experiences of feedback conversations, and their longitudinal impact, are largely invisible to educators. While we know more about what makes for an effective feedback conversation, we are yet to fully understand how learners make sense of these encounters over time. This has implications for understanding the potency of feedback on practice and for how best to support learners, particularly when the experience is challenging. Our research aimed to explore the characteristics of difficult feedback and the temporal aspects of feedback sensemaking.

**Methods:**

We collected written narratives from 32 doctors and 49 medical students via an online questionnaire about a time when they received difficult feedback in a clinical setting. Open- ended prompts facilitated participants to describe their experience, the evaluation of it over time, and the learning that arose from it. We undertook reflexive thematic analysis of the data, informed by constructivist sensemaking perspectives.

**Results:**

Difficult feedback conversations were characterized by unpredictable and complex interactions between emotions, sensemaking and learning. Despite some feedback perceived as personal, inappropriate, or threatening, feedback information was seldom disregarded. Participants engaged in feedback sharing and reflection, which often extended over years, contributing to identity work and to development of their clinical practice.

**Discussion:**

Our findings highlighted the temporal nature of sensemaking of difficult feedback and challenge expectations for learners to respond and commit to progress in the moment. Providing learners with time for reflection on feedback and opportunities to connect with personal and professional networks may facilitate the longitudinal sensemaking processes.

## Introduction

Throughout their training and beyond, medical students and doctors spend time engaging in feedback conversations with multiple stakeholders about their own performance and what they should be aspiring to achieve. Educators may facilitate effective feedback conversations by assisting the learner to be self-directed and by listening, questioning and challenging them in a supportive environment [1]. Feedback is often sifted for its quality and usefulness through the filters of the learner’s motivations [2] and interactions with peers [3]. As such, feedback is a social process, influenced by both the learner’s knowledge, attitudes and values [4], and by external forces such as social hierarchy and language use [5].

Despite years of research examining factors that promote effective feedback experiences [6], feedback often underperforms and fails to influence the learner’s performance [7]. One explanation for this is the tendency for educators to look for immediate responses and changes, when in fact it may take time and effort for learners to process and incorporate feedback into their practice [8]. This may be especially true in situations when feedback conversations challenge learners, either by stirring strong emotion or by presenting them with information that conflicts with their self-view [9]. Investigating how learners make sense of feedback conversations may contribute to building effective practices [10].

We looked to sensemaking perspectives described in the organizational psychology literature to inform our understanding of how learners process feedback. Sensemaking has been defined as ‘a process prompted by violated expectations, that involves attending to and bracketing cues in the environment, creating inter subjective meaning through cycles of interpretation and action and thereby enacting a more ordered environment from which further cues can be drawn’ [11 p67]. We drew upon the seminal work of Weick who identified that sensemaking occurs in response to uncertainty or disruption and is processed through interpretation and enactment [12]. Taken together, Maitlis and Weick’s perspectives on sensemaking provide this study with a contextual and temporal dimension to feedback, providing reasons why a person may have acted one way at one time and differently later. Their constructivist approach enabled us to view sensemaking of feedback as situational and interactive [12,13,14,15] and resonated with our understanding of feedback as relational, learner centered, temporal and actionable [16].

There is increasing awareness of the influence of emotions on sensemaking [13]. This is reflected in feedback literacy frameworks which draw attention to the role of emotions in processing feedback information. Learners must attune to and manage affect to enhance the evaluation of feedback. [17,18]. Emotions have the potential to strongly influence motivation, problem solving, professional identity development, goal setting and one’s approach to life-long learning [19,20,21]. Positive feedback experiences may motivate the learner to make changes to their practice and build collaborative relationships with educators [22]. However, when feedback threatens a learner’s self-esteem, it may be less impactful [1,23,24]. They may respond by mounting a defensive response, disengage with the process [25] or reject the feedback [10]. The work of Kluger, DeNisi [26] on Feedback Intervention Theory draws attention to such learner responses by suggesting that as the focus of feedback moves further from the task at hand and closer to the self, it becomes more difficult for individuals to use and less likely to effect a change in performance.

Sargeant et al. [27] found that around half the doctors who received negative multisource feedback did not accept or use the feedback, with some doctors experiencing lasting emotional impacts. Of these, shame has received particular attention in recent research [28,29]. Shame may be described as an ‘interruption to the experience of being, which both undermines and constitutes a sense of identity’ [30 p1136]. A discrepancy in how learners value their individual and social self may contribute to a loss of self-esteem. The effects of shame on learning extends to disengagement, avoidance behaviors and diminishment of learner physical and mental wellness [31,32].

Given the potential for feedback to illicit strong emotions, allowing time for learners to process and make sense of the information they receive is important [33], particularly when feedback is difficult or unexpected. While literature in organizational psychology [34,35] and higher education [36,37] considers the temporal relationships with feedback sensemaking, this concept has received less attention in the context of workplace medical education. Our understanding of how doctors and medical students make sense of feedback encounters and what they do with the information over time, remains unresolved. By adopting a temporal perspective in feedback research, educators may increase their understanding of the relational aspects of feedback and better trace feedback impact [38,39,40].

In our study, we aimed to investigate the characteristics and temporal aspects of challenging or difficult feedback conversations. We anticipated that such experiences are subjectively constructed and more likely to activate extended sensemaking processes. We avoided the binary term of negative feedback which could potentially be perceived by some participants as more threatening or prompt them to focus on criticism. We sought to examine a breadth of feedback experiences that spanned the educational learning continuum by including doctors and medical students, both of whom we defined as learners [41] in this study.

We asked two research questions:

1. What are the characteristics of challenging feedback encounters experienced by doctors and medical students as part of their learning trajectories?
2. How do doctors and medical students make sense of formal and informal workplace feedback conversations over time?

By developing an understanding of the sensemaking trajectory and impact of feedback experiences, we hoped to inform strategies for enhancing the learner’s capacity to grow from them.

## Methods

### Design

Our study adopts a constructivist perspective of sensemaking that emphasizes the importance of understanding human experiences within their unique contexts and recognizes the existence of multiple realities [42]. This approach acknowledges that doctors and medical students construct plausible accounts of their feedback experiences informed by their identity, interactions with others and environmental cues. As such, sensemaking is dynamic, iterative and may inform subsequent behaviors.

We chose to collect data in the form of brief written narratives through an online questionnaire, with prompts based on the structure of a narrative as proposed by Labov [43]. His framework attends to the sequence of events in a story that can assist people to make sense of their experiences [44]. Labov described six narrative categories: the *abstract*, which relates to what the story is about; the *orientation*, which contextualizes the story in person, place and time; a *complicating* action, which describes a problem experienced by the narrator; and *resolution* being a reaction to the complication. As the story unfolds, the narrator may emphasize specific points to show meaning (*evaluation*) and may signal an end to the story by returning to the present time (*coda*). Through narrative, social, cultural and institutional influences may be revealed, which shape peoples’ sensemaking [45].

In our study, we employed this framework to develop the prompts and organize the collection of data through written accounts. This method may provide several benefits for participants: it may support them to construct their thoughts chronologically; help capture a more comprehensive account of the feedback conversation, and encourage recall and reflection on the meaning of their experiences. Furthermore, the anonymity afforded by using this method may facilitate more candid responses. Rather than collecting opinions about difficult feedback, our research aim was to investigate *what* happened in the individual’s account. To this end, written narratives enabled us to address the research questions of exploring feedback characteristics and sensemaking processes over time.

We invited participants to share in writing one narrative about a time when they received feedback in a medical setting that was personally challenging or difficult. We provided the following prompts to guide their responses:

1. Briefly describe what happened in the feedback encounter (abstract/complicating action)
2. When, where and who was involved? (orientation)
3. How did this make you feel at the time and why? (evaluation)
4. How have you processed the encounter since it happened? (evaluation)
5. Has the feedback influenced your knowledge or learning strategy or performance? (resolution)
6. How do you feel now about that feedback encounter? (coda)

We also asked the participants’ gender, and for doctors, the year they graduated from medical school. The questionnaire was delivered using Qualtrics software and data was collected between May and October 2022. All participants provided informed consent before completing the questionnaire. Students received an AUD $20 gift voucher and doctors were given the opportunity to receive one of five AUD $200 gift vouchers. Ethical approval was provided from a health service (HREC/2022/QGC/86515) and a university (2022-24708-33414-6).

### Setting and Participants

We recruited medical students studying the Doctor of Medicine (MD) at an Australian university. Students were in full time clinical placement, studying at different time points in the course and geographically dispersed in metropolitan and rural locations. All students in the course undertook a vertically integrated feedback literacy program embedded in the professional practice curriculum. We disseminated information about the study to students through their online learning management system and the student society social media channels. For doctors, we recruited participants by advertising the project through several large Australian medical colleges and though professional networks.

### Data analysis

In this study we explored the characteristics and meaning of difficult feedback experiences for doctors and medical students through reflexive thematic analysis of 81 narratives [46]. Two research team members (LG, JH) initially familiarized themselves with the data set by reading all narratives. Next, all research team members (LG, JH, AR, EM, CW) reviewed ten doctor and ten medical student narratives independently to identify key characteristics in the data. The research team then met to discuss their impressions of the data. Informed by these discussions, (LG) and (JH) employed an inductive approach to analysis of the data and initially coded all narratives by assigning descriptive labels to key characteristics represented across the dataset. We met fortnightly to ensure consistency in our definition of codes, agreeing upon key examples of participants’ phrases. We then mapped conceptual similarities across the codes, categorized and generated themes. Given the prominent role of emotions highlighted in the thematic analysis, we also anlayzed shifts in valence of emotions over time. The sequencing of the narrative prompts allowed us to code for emotions described during and after the difficult feedback and at the closure of the narrative. This approach provided insights into the emotional trajectories of difficult feedback experiences and engaged us to think about sensemaking processes in the overall accounts. Throughout data analysis, the research team met quarterly. All meetings were recorded, transcribed, and used to prompt further reflexive discussions between researchers, and explore and resolve any differences in our interpretation of the findings as the project evolved.

### Reflexivity

The research team are all healthcare professionals with diverse backgrounds and expertise. (LG), (AR), (CW), are medical doctors, while (EM), and (JH), are allied health professionals. Collectively, we possess extensive experience in qualitative research and investigating health professionals’ educational practices. Reflexivity in research teams is crucial for improving the quality of the research [47] and acknowledges the dynamic and interactive process of collective and individual reflections influencing and contextualizing the outcomes of the research project [48]. As part of our study design, we piloted the survey. We distributed the survey to several colleagues in the department for feedback on the narrative prompts and completed the survey ourselves (LG, JH, AR). During the study, we reflected on our own experiences of challenging feedback encounters. We recalled experiences that had been both difficult in the moment and influential over time, which enabled us to approach the narratives with curiosity if others had experienced these shifts in perspective. This reflexivity exercise allowed us to draw upon introspection and recognise how our experiences and perspectives may influence the research process.

## Results

Thirty-two doctors and 49 medical students responded to the questionnaire. Student narratives ranged in length from 38 to 630 words (average 260 words) while doctor narratives ranged from 86 to 1153 words (average 304 words). Participants were a mix of genders (Table 1). Notably, for the medical doctors, male participants had, on average, graduated from medical school more than 10 years before female participants.

**Table 1:** Participant Characteristics.


	DOCTOR (n = 32)	MEDICAL STUDENT (n = 49)

Male	13	23

Female	19	25

Preferred not to say		1

Years since graduation		

0–5 years	3	N/A

5–10 years	3	N/A

11–20 years	6	N/A

21–30 years	9	N/A

31–40 years	8	N/A

>40 years	3	N/A


In presenting the results, we firstly share one complete narrative that illustrates the nature of our data and provides insights into the sensemaking process over time (see Table 2, below). The narrative exemplifies key themes identified across the data set which will be later described.

**Table 2:** Student Narrative Male.


**Briefly describe what happened in the feedback encounter?** I received immediate verbal feedback in the ortho *(orthopaedic)* office after being asked to present an impromptu brief surgical long case for my first time – I stumbled in delivering a structured presentation, having not yet learnt it completely, and was unsure on how to continue smoothly. The reg *(junior doctor)* sounded annoyed (though did not use degrading language) and was thorough in critiquing at length what was missing or wrong.

**When, where and who (their role) was involved?** 2020 mid-year (after lockdown 1) as MD2 *(second clinical year)* – public hospital ward office. Orthopaedic registrar giving feedback. My MD2 friend was also sitting next and listening in.

**How did this make you feel at the time and why?** I felt embarrassed as I clearly knew I did not perform to an even acceptable standard which came from under-preparedness, and I could tell the registrar picked up on that so I felt like I wasted their time with a woeful presentation. This feeling was a little accentuated with having my friend watching this. However, though there were negative feelings of inadequacy brought on within myself, I definitely felt it was challenging feedback which I needed to hear as still being valid and useful.

**How have you processed the encounter since it happened?** On the day I debriefed with my friend later outside at the clinical school – they prompted me to not dwell on the performance. I felt it was one of those ‘bad days’ that highlighted something I needed to work on.

**Has the feedback influenced your knowledge or learning strategy or performance?** Reflecting on the feedback and presentation highlighted a weak area to focus on. While COVID has got in the way, I have practised presenting cases more often and proactively especially in MD4 *(final clinical year)-* I have also sought to observe more closely how other peers and doctors presented cases.

**How do you feel now about that feedback encounter?** Two years later, I feel it was a pivotal point in MD2 which highlighted some of my learning weaknesses (presenting cases confidently and eloquently) that I needed to address more through medical school – so I don’t feel hurt or shameful about it, but I can now appreciate it from a distance.


In this narrative, a final year medical student recalled a time two years prior when he received feedback from a senior doctor on his case presentation (Abstract.) The event took place during a rotation in the orthopaedic unit, after he returned to clinical placement in the first year of the COVID pandemic (Orientation). The task was unrehearsed, this being his first attempt at learning the new skill, and the doctor’s tone used in the conversation contributed to the difficulty of the feedback encounter (Complicating actions). The student’s appraisal of his performance aligned with that of the doctor’s, whose feedback reinforced absent or incorrect aspects of his case presentation. He initially described a range of shame emotions at the time, amplified by the presence of his peer (Evaluation).

Through feedback sharing with a friend, his sensemaking indicated work to depersonalise the feedback and focus on utilisation of the feedback to improve future case presentations. Upon reflection, the student identified aspects of his case presentations that he needed to work on and made changes to his future learning behaviours (Resolution). In closing the story, the student described that with the passage of time, he no longer held shame emotions in relationship to his performance and reaffirmed the positive evaluation of the experience (Coda).

The chronological structure of the narrative provided valuable insights into the temporal process of sensemaking difficult feedback: disruption, interpretation, ordering of the experience, shifts in emotion and reframing the experience. The narrative also illustrates key themes generated in the broader data set relating to characteristics of difficult feedback described by doctors and students. These elements included the content of the feedback, the context in which it was delivered and elements like tone of the feedback provider. In this student’s account, emotions were described both during and after the feedback conversation, which stimulated engagement in reflection and feedback sharing. While the experience was remembered as painful, with adequate time for sensemaking, transformative learning was described.

Next, we share the results of the thematic analysis of the full data set, illustrating key concepts with direct quotations from doctors’ (D) and students’ (S) narratives.

### The difficulty experienced in challenging feedback

Participants described characteristics of the feedback experience that caused them to consider it challenging or difficult. These related to who provided the feedback, what the feedback was about and how the feedback delivery was experienced. Participant interactions were primarily with doctors, typically those in more senior positions (i.e., through titles such as Head of Department, as a training supervisor or through years in the profession) and, to a lesser extent, with a variety of other sources including patients, peers, and nurses.

Participants described feedback that related to clinical knowledge, skills, communication and professionalism. In the following example, a patient provided feedback to a doctor on his communication skills: ‘Weeks or months later she returned and, again, towards the end of the consultation, she fed back to me that she felt my answer was insufficient and dismissive’ (D1). The doctor was surprised by the feedback, for it challenged his perception of the patient relationship that he had established over a long period of time. He reflected upon the conversation several times and reframed the experience positively by emphasising that the patient was able to speak up and raise her concerns. The doctor was subsequently motivated to be more attuned to the patient’s needs. For one final-year student who received feedback relating to her punctuality, attendance, and examination skills, she felt ‘ashamed, because part of the feedback about punctuality was true and it confirmed all my negative self-talk that I was a ‘lazy’ medical student and not good enough’ (S16). The student used this feedback to become more punctual in subsequent rotations and on reflection, ‘felt proud’ of the changes she had made.

How doctors or students experienced the delivery of the feedback influenced their perceived difficulty of the feedback encounter. Participants used adjectives such as ‘harsh’ (S7, S16), ‘crass’ (S13), ‘blunt’ (S20, S42, D22), ‘unkind’ (S44), ‘antagonistic’ (S4) and ‘accusatory’ (S18, D32) to describe the feedback exchanges. Further, one doctor described being ‘yelled at’ (D13) while another was physically pushed aside by the feedback provider, who took over performing the task. Overwhelmingly, the participants’ narratives of their difficult feedback experience positioned feedback as telling rather than dialogue.

There were several instances whereby the feedback was perceived as gendered or personal. A female junior doctor was criticized by a male supervisor for starting a personal relationship during the rotation, while one doctor recounted an experience 10 years prior, whereby she was told she was ‘too nice to do Emergency Medicine’ and would ‘be better off with a sex change than a PhD’ if she wanted to be an Emergency physician (D14). Female doctors were also told they were ‘too middle class’ (D2) and ‘smiled too much during patient consults’ (D19). Despite the feedback relating to personal attributes or choices, some participants made changes in response to the feedback. For instance, one medical student who was told by a male doctor that she spoke like a ‘high school girl’ (S31) responded by investigating ways to modify her speech pattern.

### Difficult feedback conversations are emotional experiences

Shock and surprise were common emotions initially experienced, particularly when the feedback was unsolicited, occurred in a public forum, or was rushed or ill-timed. Students often expressed a variety of shame-related emotions, such as feeling ‘stupid’ (S9), ‘inadequate’ (S36), ‘incompetent’ (S28), or ‘like a failure’ (S42). These emotions were associated with perceived shortfalls in their clinical acumen, which appeared to threaten their professional identity. For example, one student who received feedback on his physical examination of a patient, felt ‘like I didn’t belong in medicine’ (S26) while for another student ‘initially I was quite shocked and overwhelmed following this feedback, as I have always been a very diligent and safe medical student’ (S18). Doctors also provided similar accounts of shame in their narratives; for example, one doctor stated that he felt ‘bad as a person -ashamed’ (D7) after being told ‘you should know that in your role as a doctor’ while another doctor reported feeling ‘miserable; and not good enough’ (D30) when told by another doctor that he did not advocate for a frail patient in the emergency department.

### Time for sensemaking of difficult feedback

Following difficult feedback conversations, doctors and students reflected on the experience. They often debriefed with their team, peers, colleagues, friends, and family members. Only two individuals reported writing about the feedback in personal journals. Sensemaking was an iterative process for some, occurring over days to years. After time for reflection, shifts in perspectives were noted. Despite the initial discomfort felt, most narratives concluded with a positive evaluation. For instance, one doctor explained, ‘my initial shame and dismay has moderated into gratitude for the stimulus I was given to change my behaviour’ (D3). Similarly, a student commented, ‘I now see that this interaction was essential for me in getting my head back into the medical world. I am glad that I have had experiences like this that help me to take ongoing feedback that I receive’ (S7). Through praising their own work effort, learning approach, level of skills and knowledge or professional attitudes, participants demonstrated their efforts to gain perspective on the experience. For example, ‘I have tried to focus on the truth in the feedback, even if it was given abruptly’ (S20) and ‘I received no structure on how to improve-just do better!… luckily I was someone that it spurred on to change. I asked advice from senior peers as to what to do’ (D21). More often, doctors reflected on the clinical context or feedback provider rather than their own performance when making sense of it. For example, one doctor explained: ‘I feel mildly annoyed that systems issues contributed to this and these were not acknowledged at the time’ (D16) while another attributed her difficult feedback encounter to ‘a clash of personalities and perhaps some personal issues going on with that consultant’ (D6).

However, the passage of time for some participants did not lead to a change in their appraisal of the initial feedback encounter and they still evaluated the experience negatively. This occurred in times when feedback was judged as unfair; one student who received feedback on a work-based assessment responded with a decrease in motivation to improve and after time was still ‘frustrated’ (S6) while a doctor who felt she was discriminated against for working part time, tried to move on by providing feedback to the department about the unjust working conditions.

Some doctors continued to reflect on the challenging feedback encounter long after it occurred, sometimes for years or even decades. One doctor shared that ‘Even now, almost 5 years later, I still have sudden moments where I feel bitter about it, and have to do work inside my head to address that’ (D23), while another doctor who was told that he wasn’t performing as well as his peers, stated ‘17 years later it is still as clear as day, the sick feeling that I felt at the time’ (D21). These examples illustrate for some doctors and students, the ongoing nature of the sensemaking process when difficult feedback is revisited throughout the learning journey.

### Learning from difficult feedback

In all but one narrative, doctors and students described how the difficult feedback influenced their practice. Students, for example, described improvements in their case presentations, ability to perform a skill, confidence to speak up, focus during interactions with patients and time management. For one student the experience helped him reframe the value of feedback and stated, ‘I think I am better at seeing harsh feedback as simply a way to improve next time, as opposed to an insult to my ability’ (S7). Doctors also described a range of changes in their practice in areas of documentation, clinical acumen, approach to hierarchy, patient advocacy, handover and personal interactions in the workplace. In the case of one doctor, the feedback experience was transformative and he described it as a ‘significant fork in the road’ (D3) for his professional development. He speculated on the potential cost to patients had he not made sense of the feedback experience.

In some instances, participants reported a better understanding of the role of effective feedback and indicated a commitment and motivation to improve their own feedback practices and the experiences of others. For instance, one participant stated, ‘my initial negative response has reinforced the importance of feedback delivery, and I will keep this in mind when delivering my own feedback to others’ (S5), while another drew on the feedback memory when feeling frustrated by a junior doctor to ‘calm down to give feedback in a less/non-emotionally threatening way’ (D9).

## Discussion

Our study analyzed doctor and medical student written narratives of a time when they received challenging or difficult feedback in a medical setting. The findings highlighted the complex interplay between emotions, sensemaking and learning, and the unpredictable nature of feedback impact. Participants vividly narrated emotions that they experienced during and after the difficult feedback conversation and the work that they undertook to make sense of a very challenging and memorable time during their professional practice. Participants did not disregard or casually overlook the feedback and were compelled to engage in reflection and feedback sharing, which frequently triggered learning, albeit not always in line with the feedback content.

Making sense of difficult feedback conversations was an extended process for some, notably for doctors with years of practice behind them. Our findings suggest a different view from the ‘outcome of sensemaking is that sense is restored, and at that point, sensemaking stops’ [49 p20]. Rather, some participants returned to the feedback experience during times when emotions resurfaced, when they reframed the experience as their careers evolved, or when they encountered similar experiences in their future practice or when supervising others. These findings highlight the temporal dynamics of processing feedback which is characterized by a ‘sensemaking loop of forward action and retrospective deliberation’ [13 p9].

Our findings support a relationship between participants’ emotions and the sensemaking of feedback. Emotions may serve as a trigger for initiating sensemaking while also influencing how the experience is processed [13]. The capacity for emotions to increase the intensity and meaning of the feedback process for enhancing learning was evident in our results [50]. Emotions functioned to protect, aid recovery, and connect participants with social supports and evolved over time [22]. While participants opened their stories with a distressing account of the challenging conversation, they frequently ended it by expressing gratitude for the learning outcome or towards the feedback provider. In these instances, some learners described behaviors that could be interpreted as demonstrating their self-efficacy and capacity to constructively process emotions for a positive outcome.

Both students and doctors described strong emotions, including shame [29]. While negative emotions are thought to hinder sensemaking, some of our participants who described shameful emotions also shared their story of resilience and personal growth. Our results suggest that feedback can be influential even when it challenges a learner’s self-worth, but that considerable time and sensemaking is required for this impact to materialize. While Feedback Intervention Theory [26] offers useful cautions about feedback that threatens self-esteem, its lack of attention to the temporality of sensemaking, may oversimplify the relationship between impact and risk to self. Our study emphasizes the importance of looking beyond the point of feedback provision to better understand its enduring impact.

Most participants engaged in feedback sharing with peers, family, and friends, corroborating the idea that those providing feedback are not always central to processing feedback information [36]. Our findings reinforce the idea that sensemaking is socially constructed and provides potential for an individual to change [51]. In our study, feedback sharing facilitated opportunities for participants to plan for future work, to self-evaluate and to make comparisons with their peers [19,52]. We noted potential differences in feedback sharing practices between students and doctors. We speculate that feedback sharing may be more challenging for doctors given the time pressures of clinical practice, the potential for greater threat to a well-established professional identity and the limited access that they may have to formal support structures within the clinical environment. The literature highlights that for doctors who work in a rural setting or in solo practice, there may be fewer opportunities to engage in work-based feedback conversations and they may benefit from a mentorship service to enhance their reflection on feedback [27]. Our findings indicate further insights could be gained by exploring the relationship between support mechanisms for doctors and their experience of feedback sensemaking.

Our results suggested a possible reluctance among learners to dismiss feedback, even when it was difficult. Learner hesitancy to disregard feedback information has previously been identified in a study of junior doctors working in the Emergency Department [53], regardless of how they felt about the feedback or the credibility judgement of the feedback provider, they were reluctant to throw it away. Feedback literacy is considered a graduate attribute in higher education [54] thereby setting an expectation for students to apply feedback seeking and appraisal skills. We speculate that learners may feel pressured to respond to all feedback, lest they be seen as disengaged, lacking in insight, or even unprofessional. The ability to make a distinction between useful and damaging feedback may be improved through more explicit learner orientation to what constitutes good practice. The research of Tai et al. [55] draws attention to the importance of learners developing evaluative judgement for recognising and applying standards when appraising their own work and that of others. This may be key to providing learners and seasoned doctors with more confidence to determine what feedback should be deflected and what should be acted upon.

While making sense of difficult feedback may be viewed positively, we suggest that it may, in some instances, be associated with maladaptive or damaging behaviors. We were concerned by some participants who were compelled to do something with feedback that seemed inappropriate or highly personal. Feedback that was perceived as gendered or class related raises concerns for a culture in medicine that can unconsciously promote conformity to a narrow professional identity rather than foster authenticity in the workplace. It is well recognized that prevailing gender norms and the effects of prejudices and discrimination can impact medical students’ learning and career intentions [56,57,58]. Our study highlights the negative impact of the hidden curriculum that exposes learners to outdated ideas of the medical profession [59] and that fails to challenge stereotypes. We promote the view that educators play an important role in modelling inclusivity in the profession and supporting learners to build their professional identity. For novice learners who are trying to fit into a new environment, disregarding feedback, even when potentially harmful, may be more difficult.

## Strengths and limitations

Our research should be interpreted in the context of its strengths and limitations.

We chose to survey participants and requested that they respond to six prompts designed to construct a narrative of an experience. We noted significant variability in the participants’ length of responses, which may have reflected lower engagement with the task, individual writing style or constraints from the prompt structure. Participants familiar with the function of reflective writing may have felt compelled to write a story whereby they shared a lesson learnt and represented themselves with a growth mindset. A limitation of obtaining written narratives over oral stories prevented the research team from clarifying or exploring the story in more detail,;as such the analysis may not have reflected the participant’s experience fully. Despite these limitations, the recruitment strategy enabled a diverse range of stories to be shared from participants, who spanned the length of the learning continuum, from novice through to medical experts. This enabled a greater understanding of the trajectory of sensemaking.

Although the medical students were from a single University, they represented a cross-section of individuals from three-year levels of the course and who have typically studied in several urban and rural healthcare settings. Novel to this group of students however, and which may impact the transferability of the findings, is their engagement in a feedback literacy program, which is vertically embedded in the professional practice curriculum and designed to foster reflective practice. The high response rate from students may reflect their sensitisation to the concepts of feedback literacy and a desire to share their experiences. The sensemaking of difficult feedback may be different for medical students who are less familiar with feedback literacy principles.

## Conclusion

Our research findings offer valuable insights into how doctors and medical students make sense of difficult feedback conversations. The stories highlight the breadth and capacity of emotions to influence sensemaking and the changes in thinking, knowledge and practice that can result. In the face of strong emotions, we challenge the expectation for learners to respond and commit to progress in the feedback moment. Rather, we encourage educators and supervisors to provide opportunities within the curriculum and workplace to support the longitudinal sensemaking process. By assisting learners to connect with personal and professional networks who can validate or challenge judgements, learning from difficult feedback experiences may be enhanced.
